# Inoculation of *Schizolobium parahyba* with Mycorrhizal Fungi and Plant Growth-Promoting Rhizobacteria Increases Wood Yield under Field Conditions

**DOI:** 10.3389/fpls.2016.01708

**Published:** 2016-11-22

**Authors:** Martha V. T. Cely, Marco A. Siviero, Janaina Emiliano, Flávia R. Spago, Vanessa F. Freitas, André R. Barazetti, Erika T. Goya, Gustavo de Souza Lamberti, Igor M. O. dos Santos, Admilton G. De Oliveira, Galdino Andrade

**Affiliations:** ^1^Laboratório de Ecologia Microbiana, Departamento de Microbiologia, Universidade Estadual de LondrinaLondrina, Brazil; ^2^Grupo Arboris-Manejo FlorestalDom Eliseu, Brazil

**Keywords:** microorganism interaction, reforestation, Amazon forest, *Schizolobium parahyba*, mycorrhizal inoculant

## Abstract

*Schizolobium parahyba* var. *amazonicum* (Huber ex Ducke) occurs naturally in the Brazilian Amazon. Currently, it is being planted extensively because of its fast growth and excellent use in forestry. Consequently, there is great interest in new strategies to increase wood production. The interaction between soil microorganisms and plants, specifically in the roots, provides essential nutrients for plant growth. These interactions can have growth-promoting effects. In this way, this study assessed the effect of the inoculation with arbuscular mycorrhizal fungi (AMF) and plant growth-promoting rhizobacteria (PGPR) on growth of *S. parahyba* var. *amazonicum* under field conditions. We used two native species of arbuscular mycorrhizal fungi, *Claroideoglomus etunicatum* (Ce), and *Acaulospora* sp. (Ac); two native strains of *Rhizobium* sp. (Rh1 and Rh2); and a non-native strain of *Burkholderia* sp. Different combinations of microorganisms were supplemented with chemical fertilizers (doses D1 and D2) in two planting methods, seed sowing and seedling planting. In seed sowing, the results showed that treatments with Ce/Rh1/Fertilizer D2 and Ac/No PGPR/Fertilizer D2 increased wood yield. In seedling planting, two combinations (Ac/Rh2/Fertilizer D1 and Ac/Rh1/Fertilizer D1) were more effective in increasing seedling growth. In these experiments, inoculation with AMF and PGPR increased wood yield by about 20% compared to the application of fertilizer alone.

## Introduction

The negative impacts of agro-industrial development and wood exploitation in native forest areas have encouraged the development of projects focused on reforestation with homogeneous stands or intercropped species of rapid growth and high commercial value. These strategies are directed at degraded areas with the objective of forest restoration or wood production. The family Leguminosae is one of the most representative in terms of number and frequency of plant species in the Amazon region of Brazil (Silva et al., 1988). Some tree species (nodulating and non-nodulating) of this family are used or have high potential uses for timber production (Sprent and Parsons, 2000) and land restoration (Faria et al., 2010).

*Schizolobium parahyba* var. *amazonicum* (Huber ex Ducke), belonging to the family Leguminosae and subfamily Caesalpinioideae, is a non-nodulating species native to the Amazon. It is considered an ecologically and economically important species due to its significant wood potential; its commercial potential has been exploited since the 1970s. Today, it is the native species most planted in the Brazilian states of Amazonia, Pará, Maranhão, and Rondonia, covering 87,901 ha (ABRAF, 2012). Due to its fast growth, *S. parahyba* var. *amazonicum* can reach an annual wood yield of 30 m^3^ ha^−1^ year^−1^ with 6 years of age (Carvalho, 2007). Moreover, it is considered an important species for carbon sequestration because it produces high levels of biomass in a short period of time (Siviero et al., 2008). The quality of its wood is suitable for furniture and plywood production.

The choice of plant species that are used for restoration and wood production in degraded lands represents a great challenge, because these species need to be able to survive under conditions of low soil fertility. These restrictive factors for plant growth can be attenuated by the action of efficient soil microorganisms such as plant growth-promoting rhizobacteria (PGPR) and arbuscular mycorrhizal fungi (AMF; Chaer et al., 2011). The microbial community in the soil plays an important role in the sustainability of plant communities (Andrade, 2004). The interaction between microorganisms and plants, specifically in the roots, provides for important nutritional requirements of plants and also the microorganisms associated with them. Thus, as the roots directly affect the surrounding microbial populations, the microorganisms present in the rhizosphere can also influence plant growth (Giri et al., 2005).

PGPR are microorganisms that colonize the rhizosphere and promote plant growth. Among them, the N-fixing bacteria (NFB) such as *Rhizobium* species can establish symbiosis with leguminous plant species, resulting in a beneficial interaction for plant growth. Some diazotrophic bacteria can help plant nutrition through biological fixation of N_2_ or production of phytohormones (Vessey, 2003). AMF, associated with plant roots, increase the uptake of soil inorganic nutrients, mainly P (Neumann and George, 2010). In addition, other benefits related to AMF are the stabilization of soil aggregates (Rillig, 2004), increasing resistance to water stress (Garg and Chandel, 2010) and protection against pathogens (Jung et al., 2012). In the mycorrhizosphere, the soil surrounding the roots and fungal hyphae (Artursson et al., 2006), AMF can interact with PGPR bacterial species, as well as with endophytic bacteria. Some belong to the genus *Burkholderia* (Bianciotto and Bonfante, 2002). These interactions can provide potential benefits for plant development. The inoculation of compatible combinations of PGPR and AMF in forest and agricultural systems may result in a significant increase in plant growth (Biró et al., 2000; Nadeem et al., 2014; Hashem et al., 2016). Many studies (Marques et al., 2001; Valdenegro et al., 2001; Patreze and Cordeiro, 2004) have demonstrated the synergistic effect of the inoculation of *Rhizobium* and AMF in promoting nodulated legume tree species. However, little research has been carried out on this subject with legume trees of the subfamily Caesalpinoideae (Bryan et al., 1996) with the formation of nodules observed in a few cases (Sprent, 1983).

*S. parahyba* var. *amazonicum* is a non-nodulating legume, and *Rhizobium* bacteria may promote plant growth in this species in two ways. Some authors suggest that non-nodulating species of the family Leguminoseae can profit from N fixed by root-associated bacteria (rhizosphere bacteria or endophytes) like nodulating species (Bryan et al., 1996; Van Sambeek et al., 2007). On the other hand, it can be assumed that the *Rhizobium* act as plant growth-promoting bacteria in the rhizosphere and release phytohormones (Mehboob et al., 2012).

The use of growth-promoting microorganisms in *S. parahyb*a var. *amazonicum* was assessed by Siviero et al. (2008), who showed that this species displays a positive response to inoculation with AMF in combination with N-fixing bacteria isolated from another plant species (exogenous, i.e., non-native bacteria). The authors observed differences between planting methods (seeds or seedlings) in inoculated plants. In the planting method with seeds, only AMF (*Glomus intrarradices*) inoculation increased biomass and wood production. In the planting method with seedlings, the dual inoculation of AMF (*Glomus clarum*) and PGPR (LEM6 or Rhi1 *Rhizobium* strains) was more effective. In this work, the authors suggested that the selection of native microorganisms is very important to obtain the best results in the field.

Our hypothesis was that the inoculation with indigenous microorganisms is more effective in promoting plant growth of *S. parahyba* var. *amazonicum*, and that the presence of inoculum would help plant roots to be more effective in using the chemical fertilizer applied. Therefore, this study assessed the effect on wood production, comparing inoculation with two indigenous AMF (*Claroideoglomus etunicatum* and *Acaulospora* sp.) isolated from *S. parahyba* var. *amazonicum* roots in interaction with three bacterial strains (two indigenous *Rhizobium* spp. and one exogenous *Burkholderia* sp.). The inoculation with different combinations of microorganisms and the addition of chemical fertilizer was investigated using a completely randomized block experiment. The effect of these factors on *S. parahyba* var. *amazonicum* growth was determined *in situ* over 2 years.

## Materials and methods

### Experimental field

The experiments were conducted in the municipality of Dom Eliseu – Pará State (Brazil) [4°17′36″ S and 47°33′15″ W]. Its climate is classified as humid mesothermic, with an average annual temperature of 25°C and annual rainfall of 2500 mm. The region in the wet season shows extensive rain from January to June, and a relative humidity of around 85%. The vegetation is a *terra firme* type with dense forest (da Silva et al., 2011). However, continuous deforestation had destroyed the original vegetation, leading to the emergence of large areas of savannas and secondary forest (SEICOM, 2012).

The soil in the experimental area was a Xanthic Ferralsol according to the FAO classification (FAO, 1994). Prior to experimentation, the soil was chemically analyzed using a composite sample collected from a depth of 0–20 cm and the physical–chemical analysis showed the following results: pH (CaCl_2_) 4.8, H^+^Al 2.9 cmol_c_ dm^−3^, Al^+3^ 0.2 cmol_c_ dm^−3^; Ca^+2^ 3.3 cmol_c_ dm^−3^, Mg^+2^ 1.0 cmol_c_ dm^−3^, K^+^ 0.24 cmol_c_ dm^−3^; P (Mehlich I) 10.0 cmol_c_ dm^−3^, C 19.0 g dm^−3^; S-${\text{SO}}_{4}^{-2}$ 4.2 cmol_c_ dm^−3^, Na^+^ 4.0 cmol_c_ dm^−3^, B 0.3 cmol_c_ dm^−3^, Fe^2+^ 99.0 cmol_c_ dm^−3^, Mn 7.3 cmol_c_ dm^−3^, Cu 0.2 cmol_c_ dm^−3^, and Zn 3.0 cmol_c_ dm^−3^. Samples of soil collected in the experimental area showed a low number of AMF spores (3 spores/g of soil) when compared with other soils.

### Experimental design

Two experiments were conducted, each using different planting methods, seeds, and seedlings. The inoculation of each planting method occurred by using different combinations of two species of AMF (*C. etunicatum* and *Acaulospora* sp.), and three PGPR strains (*Rhizobium* sp1, *Rhizobium* sp2, and *Burkholderia* sp.). Additionally, two doses of chemical fertilizer (NPK formulation 10:20:20-N: Urea; P: P_2_O_5_; K: K_2_O) were applied. Dose 1 (D1) was 75 g of fertilizer per plant and dose 2 (D2) was 150 g of fertilizer per plant. The resulting 36 treatments of combinations of these three factors (AMF, PGPR, and Fertilizer) are described in Table 1. The treatments were arranged as a completely randomized block design with three repetitions. In the block, each treatment was represented by a row with 10 plants. The spacing was 3 × 2 m between plants and 6 m between blocks. The buffer area in the experiment was composed of three rows with non-inoculated and non-fertilized plants.

**Table 1:** **Description of treatments**.

**Treatment**	**Description**
T1	No AMF/No PGPR/No fertilizer
T2	No AMF/No PGPR/Fertilizer D2
T3	No AMF/No PGPR/Fertilizer D1
T4	No AMF/Burk/No fertilizer
T5	No AMF/Burk/Fertilizer D2
T6	No AMF/Burk/Fertilizer D1
T7	No AMF/Rh1/No fertilizer
T8	No AMF/Rh1/Fertilizer D2
T9	No AMF/Rh1/Fertilizer D1
T10	No AMF/Rh2/No fertilizer
T11	No AMF/Rh2/Fertilizer D2
T12	No AMF/Rh2/Fertilizer D1
T13	Ac/No PGPR/No fertilizer
T14	Ac/No PGPR/Fertilizer D2
T15	Ac/No PGPR/Fertilizer D1
T16	Ac/Burk/No fertilizer
T17	Ac/Burk/Fertilizer D2
T18	Ac/Burk/Fertilizer D1
T19	Ac/Rh1/No fertilizer
T20	Ac/Rh1/Fertilizer D2
T21	Ac/Rh1/Fertilizer D1
T22	Ac/Rh2/No fertilizer
T23	Ac/Rh2/Fertilizer D2
T24	Ac/Rh2/Fertilizer D1
T25	Ce/No PGPR/No fertilizer
T26	Ce/No PGPR/Fertilizer D2
T27	Ce/No PGPR/Fertilizer D1
T28	Ce/Burk/No fertilizer
T29	Ce/Burk/Fertilizer D2
T30	Ce/Burk/Fertilizer D1
T31	Ce/Rh1/No fertilizer
T32	Ce/Rh1/Fertilizer D2
T33	Ce/Rh1/Fertilizer D1
T34	Ce/Rh2/No fertilizer
T35	Ce/Rh2/Fertilizer D2
T36	Ce/Rh2/Fertilizer D1

*AM fungi: Ac, Acalospora sp.; Ce, Claroideoglomus etunicatum. PGPR: Burk, Burkholderia sp.; Rh1, Rhizobium sp1; Rh2, Rhizobium sp2*.

### Plant inoculation

The seeds of *S.parahyba* var. *amazonicum* were collected from native forest in Pará state, where the tree occurs naturally. Before sowing, the seeds were scarified mechanically at one end. In the seed system, two seeds were sown in each pit and in the seedling system, one 30 day-old seedling (cultivated in a nursery in plastic bags of 1000 mL with non-sterile soil) was planted before being taken out of the plastic bag.

### Microorganism strains and growth conditions

Spores of AMF (*C. etunicatum* and *Acaulospora* sp.) were isolated from the rhizosphere of *S. parahyba* var. *amazonicum* in the Amazon Forest in Dom Eliseu, Pará, and propagated in pots with *Urochloa decumbens* as plant host. Ten grams of inoculum extracted from pots containing 50 spores/g of soil, colonized roots, and mycelia were added before seed sowing or seedling planting in the field.

The bacterial strains used as inoculum were two native ones [*Rhizobium* sp1 (Rh1) and *Rhizobium* sp2 (Rh2)] isolated from roots of *S. parahyba* var. *amazonicum* in the Amazon Forest in Dom Eliseu, Pará. In addition, an exogenous strain of *Burkholderia* sp. was used (Raimam et al., 2007). The *Rhizobium* strains were grown in Petri dishes with TY medium (Beringer, 1974) and the *Burkholderia* sp. strain in Nfb medium (Döbereiner and Day, 1976). For inoculation in the field, the bacteria were re-suspended in sterile saline (0.85% NaCl) plus carboxymethyl cellulose (0.1%) and adjusted by visual comparison with a McFarland standard scale to obtain a final cell concentration of ~10^9^ cells mL^−1^. Before sowing, the seeds were inoculated by immersion in a bacterial suspension. Seedlings were inoculated with 10 mL of bacterial suspension around the plant.

### Data collection, biomass, and wood yield determination

Plant growth was determined by shoot diameter (at soil surface), shoot total height (TH), and biomass. Data were collected at 180, 280, 480, and 720 days after planting. At 720 days, we evaluated the diameter at breast height (DBH), TH, and height up to the first leaf (HFL). Biomass (BIO) was determined as described below and was determined for each plant based on the volume of the stem (Brow, 1997) and multiplied by the correction factor for *S. parahyba* var. *amazonicum* as suggested by Colpini et al. (2009).

BIO = [π(DBH/2)HFL) × (0.7)]

Wood yield was determined by BIO-value multiplied by the wood specific density of *S. parahyba* var. *amazonicum* and the number of plants per hectare.

$$\begin{array}{l} \text{Wood yield }\left({\text{m}}^{3}\text{ h}{\text{a}}^{-1}\right)=\text{BIO }\left(0.39\right)\;\times \text{ d};\text{ where d is plants h}{\text{a}}^{-1} \end{array}$$

### Statistical analysis

Statistical analysis was performed using Statistica 7.0 (Statsoft Inc Statistica, 2004). Data were tested for normality using the Shapiro-Wilk test. ANOVA on the data sets (DBH, TH, and biomass) was carried out to determine the interactions of the factors AMF, PGPR, and Fertilizer. Differences between treatments were determined by Tukey's means test (HSD) at *p* ≤ 0.05 significance level. A principal component analyses (PCA) was carried out with all data. Time was considered a cofactor, and the treatments were grouped according to AMF inoculation to facilitate the interpretation.

## Results

### Seed experiment

**Figure 1:**
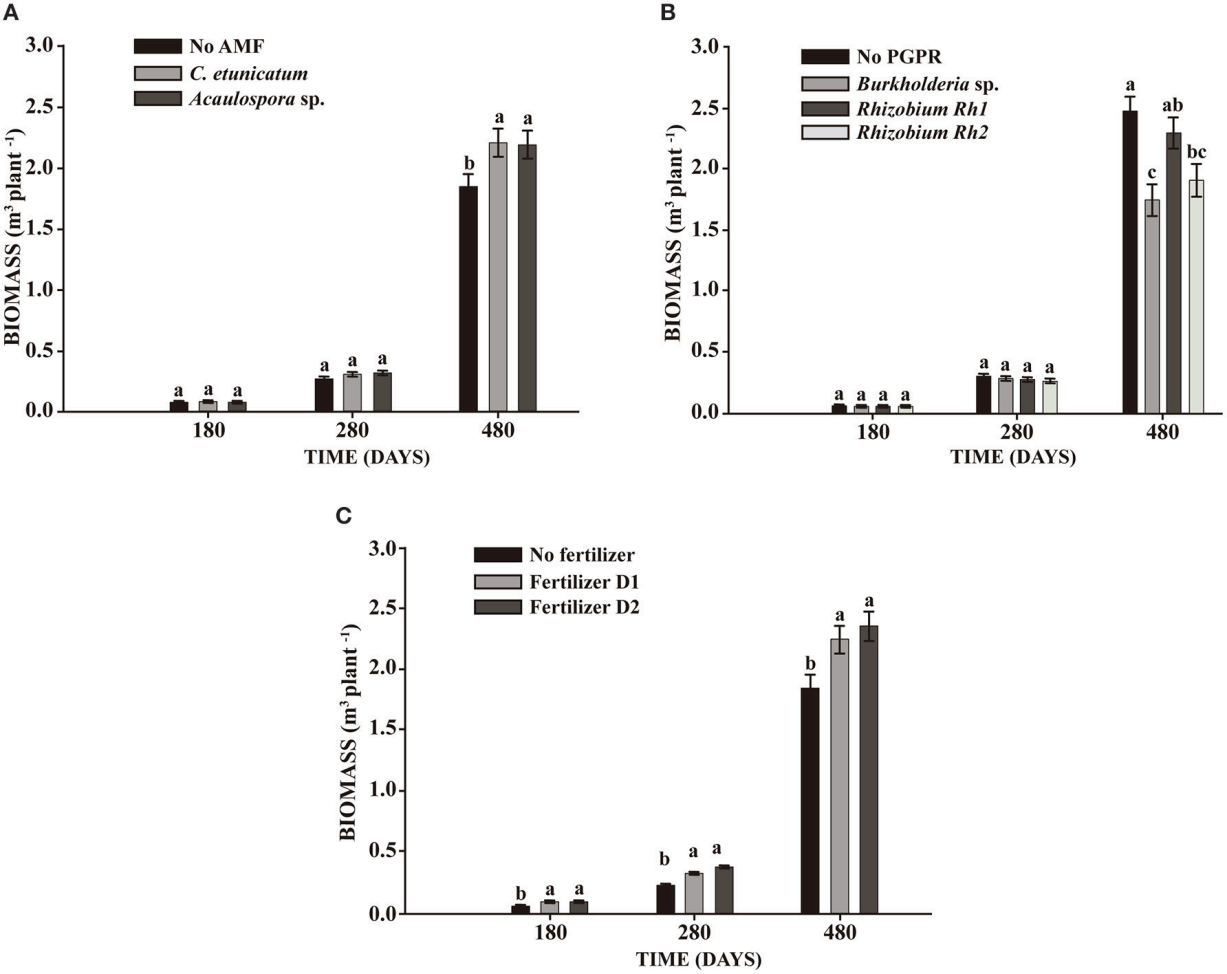
**Effect on biomass production of ***S. parahyba*** var. ***amazonicum*** after 180, 280, and 480 days of sowing seeds. (A)** Arbuscular mycorrhiza fungi (AMF) *C. etunicatum* and *Acaulospora* sp.; **(B)** PGPR strains *Burkholderia* sp., *Rhizobium* Rh1, and *Rhizobium* Rh2; **(C)** two doses of fertilizer (D1: 75 g plant^−1^ and D2: 150 g plant^−1^). Bars sharing the same letter are not statistically significantly different according to Tukey test (*p* < 0.05).

In the seeds planting experiment, AMF showed significant effects on DBH and TH at 280 days and BIO at 480 days. PGPR increased DBH and TH at 480 days. Fertilizer addition showed a significant effect on plant growth at all sampling times. The interaction between PGPR and fertilizer effect resulted in increased DBH (Table 2). BIO was significantly enhanced by both AMF *C. etunicatum* and *Acaulospora* sp. at 480 days (Figure 1A). *Rhizobium* strain Rh1 increased BIO by around 30% when compared with *Burkholderia* (Figure 1B). Both doses of fertilizer increased plant growth during the whole experiment (Figure 1C).

**Table 2:** **Analysis of variance of plant growth of ***S. parahyba*** var. ***amazoicum*** at 180, 280, and 480 days after sowing seeds**.

**FACTOR**	**(*****p*****-values)**								
	**Shoot Diameter**			**Total Height of Plant**			**Biomass**		
	**180 days**	**280 days**	**480 days**	**180 days**	**280 days**	**480 days**	**180 days**	**280 days**	**480 days**
AMF	0.167	0.016	0.046	0.072	0.030^*^	0.514	0.718	0.118	0.030^*^
PGPR	0.167	0.240	0.000^*^	0.371	0.873	0.001^*^	0.734	0.434	0.000^*^
Fertilizer	0.000^*^	0.000^*^	0.000^*^	0.000^*^	0.001^*^	0.843	0.000^*^	0.000^*^	0.000^*^
AMF^*^PGPR	0.211	0.405	0.396	0.641	0.897	0.628	0.469	0.315	0.474
AMF^*^Fertilizer	0.167	0.493	0.745	0.211	0.324	0.993	0.069	0.326	1.000
PGPR^*^Fertilizer	0.032^*^	0.019^*^	0.015^*^	0.303	0.192	0.513	0.057	0.021^*^	0.092
AMF^*^PGPR^*^Fertilizer.	0.569	0.919	0.360	0.914	0.921	0.861	0.430	0.637	0.400

*AMF, arbuscular mycorrhizal fungi; PGPR, plant growth-promoting rhizobacteria; Fertilizer*.

* *Significant difference according to ANOVA (p < 0.05)*.

After 720 days, there was a significant effect on DBH and BIO in AMF plants from sown seeds and in fertilized plants. Bacterial inoculation, especially with *Rhizobium* Rh1, increased DBH, TH, and BIO. The interaction between AMF and bacteria also increased DBH, TH, and BIO. The interaction between *Acaulospora* sp. and *Rhizobium* Rh1 resulted in greater diameter and height of *S. parahyba* in fertilized and non-fertilized plants. On the other hand, the same treatment increased BIO but only in non-fertilized plants (Table 3). The interaction between *Rhizobium* Rh1 and *C. etunicatum* also increased DBH and BIO in non-fertilized and D2 plants, and TH was increased only in D2 plants in the interaction of *C. etunicatum* and *Rhizobium* Rh1 (Table 3).

Table 3**Effect of arbuscular mycorrhizal fungi (AMF) ***Acaulospora*** sp. (Ac), ***Claroideoglomus etunicatum*** (Ce), and PGPR strains ***Burkholderia*** sp. (Burk), ***Rhizobium*** Rh1 and Rh2 on diameter at breast height (DBH), total height (TH), and biomass (BIO) after 2 years of sowing seeds**.

**Table:** 

**ANOVA**							
**FACTOR**	**df**	**DBH**		**TH**		**BIO**	
		***F***	***P-value***	***F***	***P-value***	***F***	***P-value***
AMF	2	7.39	0.0007^*^	2.50	0.0830	5.69	0.0036^*^
PGPR	3	9.33	0.0000^*^	4.75	0.0028^*^	9.26	0.0000^*^
Fertilizer	2	3.20	0.0416^*^	2.00	0.1361	3.66	0.0265^*^
AMF^*^ PGPR	6	5.18	0.0000^*^	3.80	0.0010^*^	4.26	0.0003^*^
AMF^*^Fertilizer	4	2.23	0.0646	3.35	0.0101^*^	1.76	0.1350
PGPR^*^Fertilizer	6	1.60	0.1430	0.84	0.5336	1.57	0.1515
AMF^*^PGPR^*^ Fertilizer	12	0.59	0.8454	1.22	0.2651	0.59	0.8451

**Table:** 

**MEAN TEST**									
**NFB**	**Chemical Fertilizer/AM**								
	**No chemical fertilizer**			**Chemical fertilizer D1**			**Chemical fertilizer D2**		
	**No AM**	**Ac**	**Ce**	**No AM**	**Ac**	**Ce**	**No AM**	**Ac**	**Ce**
**DBH (cm)**									
No NFB	9.79 A,a	9.98 A,a,b	11.23 A,a	11.06 A,a	11.87 A,a	11.64 A,a	10.77 A,a	12.33 A,a	11.72 A,a
Burk	9.48 A,a	8.58 A,b	10.06 A,a	9.60 A,a	9.32 A,b	10.44 A,a,b	8.72 A,b	9.97 A,a	9.67 A,a
Rh1	8.78 B,a	10.83 A,a	10.99 A,a	8.26 B,a	11.79 A,a	10.80 A,b,a	6.51 B,b	11.24 A,a	12.6 A,a
Rh2	9.55 A,a	8.59 A,b	9.32 A,a	9.84 A,a	9.96 A,a,b	8.94 A,b	9.56 A,a	10.86 A,a	9.82 A,a
**TH (m)**									
No NFB	8.35 A,a	7.92 A,a,b	8.61 A,a	9.00 A,a	9.49 A,a	8.00 A,a	8.71 A,a	9.35 A,a	9.16 A,A,b
Burk	8.10 A,a	7.12 A,b	8.26 A,a	8.00 A,a,b	7.72 A,b	8.42 A,a	6.78 A,b	8.06 A,a	7.70 A,b
Rh1	7.54 B,a	9.02 A,a	8.40 B,a	6.44 B,b	9.48 A,a	8.28 A,b,a	6.13 B,b	8.66 A,a	10.16 A,a
Rh2	8.32 A,a	7.19 A,a,b	7.50 A,a	8.28 A,a,b	8.32 A,a,b	7.88 A,a	7.12 A,A,b	8.71 A,a	8.10A,A,b
**BIO dm**^(3)^									
No NFB	57.94 A,a	61.36 A,a,b	76.72 A,a	74.04 A,a	91.20 A,a	82.76 A,a	65.75 A,a	102.09 A,a	83.69 A,a
Burk	58.39 A,a	40.63 A,b	64.91 A,a	49.79 A,a	51.84 A,b	70.36 A,a	47.24 A,a	57.71 A,b	53.01 A,a
Rh1	40.92B,a	75.33 A,a	76.28 A,a	57.42 A,a	91.49 A,a	70.37 A,a	42.94 B,a	80.68 A,b,b	103.5 A,a
Rh2	62.88 A,a	41.37 A,b	48.28 A,a	54.19 A,a	65.28 A,a,b	48.56 A,a	54.16 A,a	74.10 A,a,b	54.67 A,a

* *Significantly different according to ANOVA (p < 0.05). Means sharing the same letter are not significantly different according to Tukey HSD test (P < 0.05). Capital letters refer to comparisons of AM fungi at each dose of fertilizer (rows), and the small letters refer to comparisons between bacterial treatments (columns)*.

**Figure 2:**
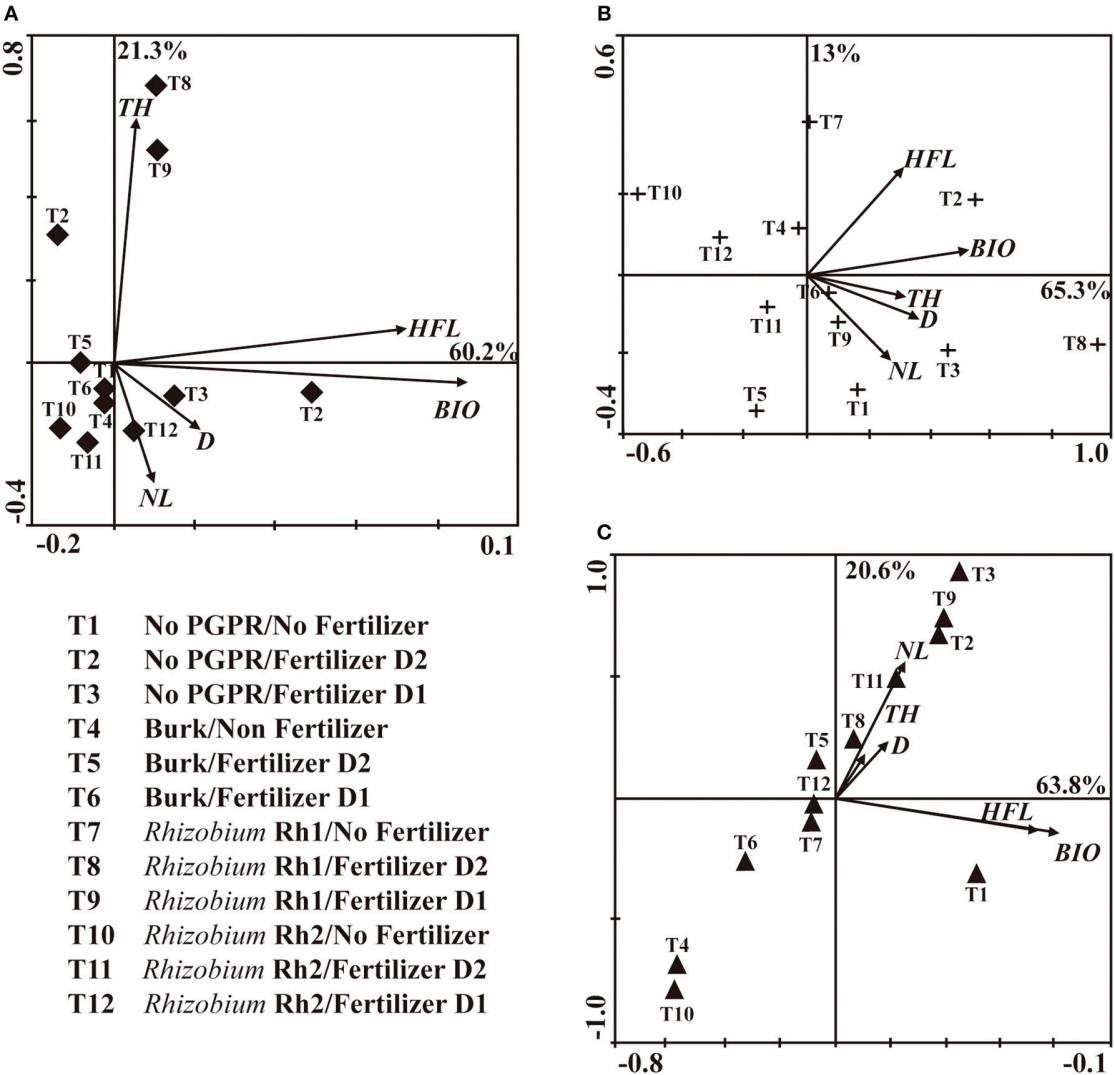
**Principal component analysis (PCA) among AM fungi and PGPR bacteria (***Burkholderia*** sp, Rhizobium (Rh1 and Rh2) on the shoot diameter (D), total height (TH), height at the first leave (HFL), number of leaves (NL) and biomass (BIO) two years after seeds sowing**. **(A)** No AMF; **(B)** *Claroideoglomus etunicatum*; **(C)** *Acaulospora* sp.

PCA of No AMF plants (Figure 2A) revealed that principal component 1 (PC1) and principal component 2 (PC2) accounted for 60.2 and 21.3% of the data variation, respectively. PC1 comprised treatments with *Rhizobium* Rh1 and fertilizer (D1 and D2), and it showed a strong relation with TH, when compared with plants that were only fertilized. In PC2, D2 showed more influence on HFL and BIO. For plants inoculated with *C. etunicatum* (Figure 2B), PC1 accounted for 65.3% of data variation and PC2 for 13%. PC1 allowed comparison of two treatments: *Rhizobium* Rh1/No Fertilizer and No PGPR/Fertilizer D2. Both treatments showed more influence on HFL and BIO. PC2 showed that the combination of *Rhizobium* Rh1 and fertilizer D2 had a significant influence on BIO. With regard to inoculation with *Acaulospora* sp., PC1 accounted for 63.8% of data variation and PC2 for 20.4 % (Figure 2C). The treatments that showed the highest impact on TH and DBH were fertilizer D1 plus *Rhizobium* Rh1, and the application of D1 or D2 as well. This analysis showed that the inoculation with microorganisms was compatible with the application of fertilizer and that it had a significant effect on plant growth. Therefore, the more effective treatments were: No AMF/*Rhizobium* Rh1/Fertilizer D2; *C. etunicatum*/*Rhizobium* Rh1/No fertilizer; *C. etunicatum*/No PGPR/Fertilizer D2; *Acaulospora* sp./No PGPR/Fertilizer D1 and *Acaulospora* sp./*Rhizobium* Rh1/Fertilizer D2 compared to control plants.

**Figure 3:**
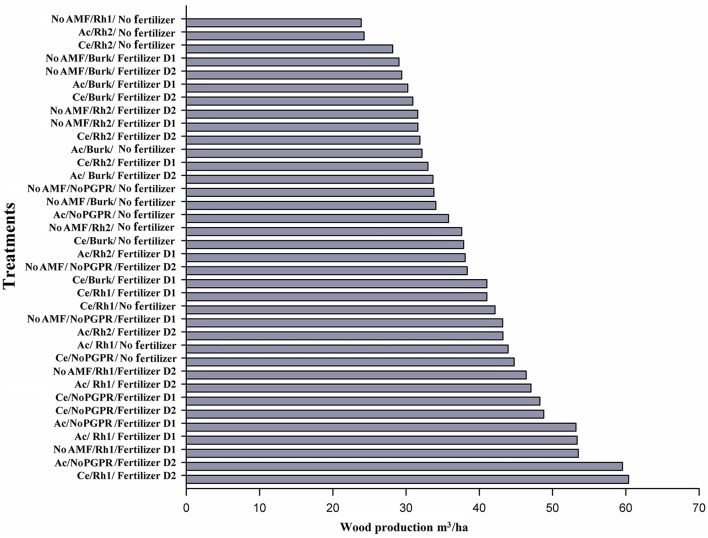
**Wood production by different combinations of AMF [***Acaulospora*** sp. (Ac) and ***Claroideoglomus etunicatum*** (Ce)], PGPR [***Burkholderia*** sp. (Burk), ***Rhizobium*** Rh1, and ***Rhizobium*** Rh2], and chemical fertilizer [D1: 75 g plant^**−1**^, D2: 150 g plant^**−1**^] 2 years after sowing seeds**.

In terms of wood yield in plants from sown seeds, only Ac/No PGPR/No fertilizer was more effective (up to 20% increase in wood production) compared control plants (No AMF, No PGPR, No Fertilizer). In addition, four treatments increased wood yield by 30% (No AM/Rh2/No fertilizer; Ce/*Burkholderia* (Burk)/No fertilizer; Ac/Rh2/Fertilizer D1; and No AMF/No PGPR/Fertilizer D2), and in seven treatments, there was a more than 40% increase (Ce/Burk/Fertilizer D1; Ce/Rh1/Fertilizer D1; Ce/Rh1/No fertilizer; No AM/No PGPR/Fertilizer D1; Ac/Rh2/Fertilizer D2; Ac/Rh1/No fertilizer; and Ce/No PGPR/No fertilizer). Four treatments increased wood yield by more than 50% (No AM/Rh1/Fertilizer D2; Ac/Rh1/Fertilizer D2; Ce/mon-PGPR/Fertilizer D1; Ce/No PGPR/Fertilizer D2), and three treatments by more than 60% (Ac/No PGPR/Fertilizer D1); Ac/Rh1/Fertilizer D1; and No AMF/Rh1/Fertilizer D1). Two treatments increased wood yield by 100% (Ac/No PGPR/Fertilizer D2 and Ce/Rh1/Fertilizer D2) (Figure 3), when compared to the control.

The presence of AM fungi was more effective by up to 40% with *C. etunicatum* (Ce/non PGPR/No Fertilizer). On the other hand, when PGPR alone were used for inoculation, wood yield increased only by 30% in the presence of *Rhizobium* Rh2, and with other PGPR no effect was observed. When yield assessed, with increasing more than 50%, the addition of fertilizer (D1 or D2) was needed as well as AMF (*Acaulospora* or *C. etunicatum*).

### Seedling experiment

**Figure 4:**
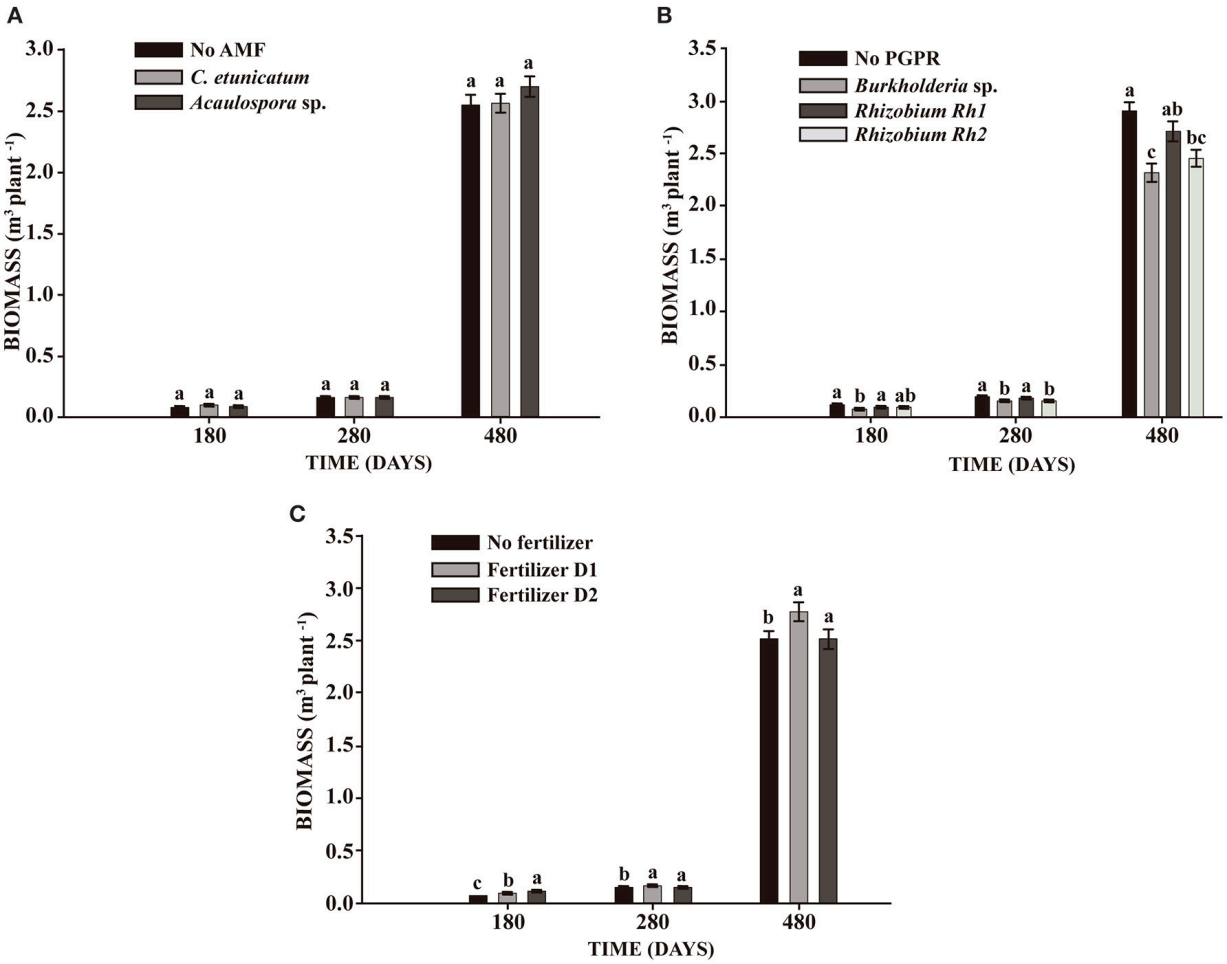
**Effect on biomass production of ***S. parahyba*** var. ***amazonicum*** 180, 280, and 480 days after seedling planting. (A)** Arbuscular mycorrhiza fungi (AMF) *C. etunicatum* and *Acaulospora* sp.; **(B)** PGPR strains *Burkholderia* sp., *Rhizobium* Rh1, and *Rhizobium* Rh2; **(C)** two doses of fertilizer (D1: 75 g plant^−1^ and D2: 150 g plant^−1^). Bars sharing the same letter are not statistically significantly different according to Tukey test (*p* < 0.05).

In the experiment with seedlings, mycorrhizal inoculation showed a significant effect on TH after 280 days. The inoculation of PGPR showed significant differences in all parameters assessed, increasing plant growth at 180, 280, and 480 days. The interaction between AMF and fertilizer showed significant differences in DBH during the whole experiment and in TH and BIO at 180 and 280 days (Table 4). No difference in BIO was observed between AMF and control plants (Figure 4A). On the other hand, inoculation of *Rhizobium* Rh1 increased BIO (Figure 4B). The addition of fertilizer showed a positive effect on BIO at all times evaluated (Figure 4C).

**Table 4:** **Analysis of variance of plant growth of ***S. parahyba*** var. ***amazoicum*** at 180, 280, and 480 days after seedling planting**.

**FACTORS**	**(*****p*****-values)**								
	**Shoot diameter**			**Total height of plant**			**Biomass**		
	**180 days**	**280 days**	**480 days**	**180 days**	**280 days**	**480 days**	**180 days**	**280 days**	**480 days**
AMF	0.856	0.890	0.424	0.384	0.027^*^	0.225	0.246	0.799	0.368
PGPR	0.000^*^	0.000^*^	0.000^*^	0.000^*^	0.000^*^	0.035^*^	0.000^*^	0.000^*^	0.000^*^
Fertilizer	0.000^*^	0.000^*^	0.009^*^	0.000^*^	0.000^*^	0.001^*^	0.000^*^	0.001^*^	0.037^*^
AMF^*^PGPR	0.030^*^	0.456	0.066	0.222	0.383	0.114	0.101	0.641	0.055
AMF^*^Fertilizer	0.128	0.153	0.044^*^	0.112	0.139	0.012^*^	0.542	0.151	0.015^*^
PGPR^*^Fertilizer	0.000^*^	0.001^*^	0.029^*^	0.020^*^	0.000^*^	0.684	0.031^*^	0.004^*^	0.061
AMF^*^PGPR^*^Fertilizer	0.606	0.489	0.095	0.975	0.580	0.066	0.881	0.694	0.259

*AMF, arbuscular mycorrhizal fungi; PGPR, plant growth-promoting rhizobacteria; Fertilizer*.

* *Significant difference according to ANOVA (p < 0.05)*.

Two years (720 days) after planting and inoculated with AMF and PGPR and/or addition of fertilizer, trees in this experiment showed elevated DBH, TH, and BIO values. Plant growth increased in non-fertilized plant inoculated with *Rhizobium* Rh1 and *C. etunicatum* when compared with No AMF plants. D1-fertilized plants plus *Acaulospora* sp. and *Burkholderia* sp. or *Rhizobium* Rh1 increased TH. BIO was increased in non-fertilized plants when inoculated with *C. etunicatum* and *Rhizobium* Rh1, and in plants fertilized with D1 plus inoculated with *Acaulospora* sp. and *Rhizobium* Rh1 (Table 5).

Table 5**Effect of AMF ***Acaulospora*** sp. (Ac), ***Claroideoglomus etunicatum*** (Ce), and PGPR strains ***Burkholderia*** sp. (Burk), ***Rhizobium Rh1*** and ***Rh2*** on diameter at breast height (DBH), total height (TH), and biomass (BIO) 2 years after seedling planting**.

**Table:** 

**ANOVA**							
**FACTOR**	**df**	**DBH**		**TH**		**BIO**	
		***F***	***P-value***	***F***	***P-value***	***F***	***P-value***
AMF	2	0.08	0.9156	0.85	0.7036	0.13	0.8749
PGPR	3	7.10	0.0001^*^	10.72	0.0000^*^	7.37	0.0001
Fertilizer	2	7.36	0.0007^*^	7.71	0.0005^*^	6.65	0.0014
AMF^*^ PGPR	6	0.58	0.7397	0.20	0.9768	0.38	0.8892
AMF^*^Fertilizer	4	1.89	0.1102	3.76	0.0049^*^	4.03	0.0031
PGPR^*^ Fertilizer.	6	1.62	0.1385	3.35	0.0029^*^	2.10	0.0511
AMF^*^PGPR^*^Fertilizer	12	1.52	0.1098	2.25	0.0086^*^	1.71	0.0606

**Table:** 

**MEAN TEST**									
**NFB**	**Fertilizer dose/AM1f**								
	**No Fertilizer**			**Fertilizer D1**			**Fertilizer D2**		
	**No AMF**	**Ac**	**Ce**	**No AMF**	**Ac**	**Ce**	**No AMF**	**Ac**	**Ce**
**DBH (cm)**									
No PGPR	11.042 A,b	11.12 A,a	10.67 A,A,b	11.13 A,a	11.28 A,a	11.84 A,a	11.22 A,a	10.80 A,a	11.01 A,a
Burk	9.36 A,b	10.17 A,a	10.53 A,A,b	10.86 A,a	11.17 A,a	10.02 A,b	9.70 A,a	9.60 A,a	10.77 A,a
Rh1	11.92 A,a	10.39 B,a	11.60 A,a	11.32 A,a	11.98 A,a	10.87 A,A,b	10.83 A,a	10.60 A,a	10.80 A,a
Rh2	10.30 A,A,b	10.01 A,a	9.34 A,b	10.76 A,a	12.02 A,a	11.41 A,A,b	10.42 A,a	10.49 A,a	11.15 A,a
**TH (m)**									
No PGPR	8.71 A,A,b	9.41 A,a	8.38 A,A,b	9.03 A,a	9.16 A,a	9.64 A,a	10.17 A,a	9.28 A,a	9.50 A,a
Burk	7.64 A,b	8.12 A,a	8.05 A,b	9.26 A,b,a	9.58 A,a	8.28B,a	7.75 A,c	7.80 A,b	8.83 A,a
Rh1	9.72 A,a	8.61 A,a	9.65 A,a	8.67 B,a	10.0 A,a	8.57 B,a	9.37 A,A,b	9.05 A,A,b	9.58 A,a
Rh2	8.42 A,A,b	8.23 A,a	7.70 A,b	9.11 A,a	9.71 A,a	8.78 A,a	8.76 A,b,c	8.61 A,b	9.38 A,a
**BIO (dm**^3^**)**									
No PGPR	79.56 A,A,b	85.27 A,a	64.31 A,A,b	83.29 A,a	90.90 A,a	93.11 A,a	93.74 A,a	75.19 A,a	93.82 A,a
Burk	49.67 A,b	55.23 A,a	68.73 A,A,b	82.13 A,a	85.49 A,a	66.38 A,a	52.73 A,b	60.61 A,a	72.93 A,a
Rh1	98.73 A,a	65.25 B,a	93.90 A,a	77.07 B,a	101.2 A,a	67.00B,a	76.39 A,A,b	75.17 A,a	79.92 A,a
Rh2	63.39 A,b	59.04 A,a	55.24 A,b	74.66 A,a	103.6 A,a	79.27 A,a	70.86 A,A,b	66.60 A,a	85.72 A,a

* *Significantly different according to ANOVA (p < 0.05). Means sharing the same letter are not significantly different according to Tukey HSD test (P < 0.05). Capital letters refer to comparisons of AM fungi at each dose of fertilizer (rows), and the small letters refer to comparisons between bacterial treatments (columns)*.

**Figure 5:**
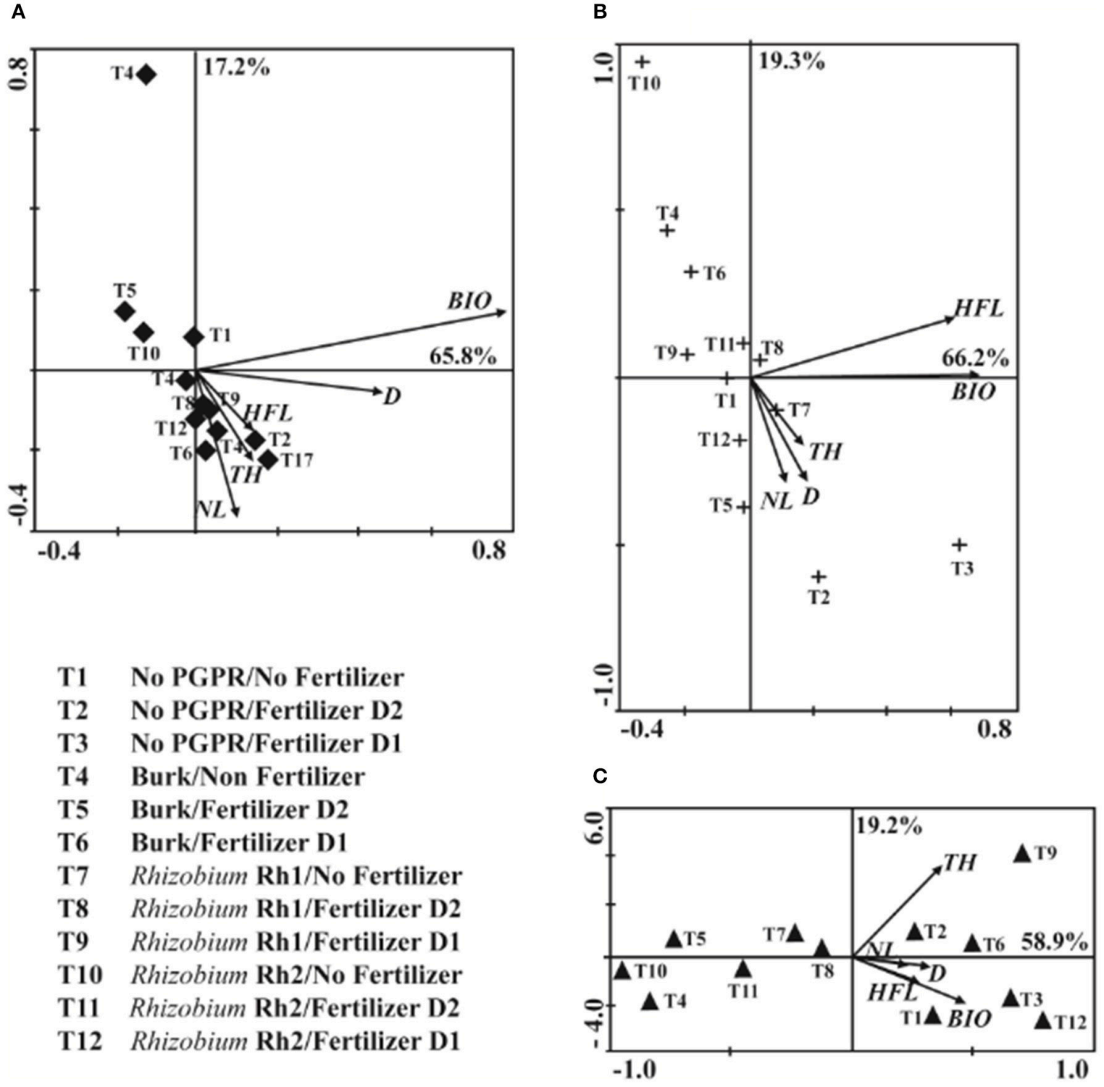
**Principal component analysis (PCA) among AM fungi and PGPR bacteria (***Burkholderia*** sp, Rhizobium Rh1 and Rh2) on the shoot diameter (D), total height (TH), height at the first leave (HFL), number of leaves (NL) and biomass (BIO) two years after seedling planting**. **(A)** No AMF; **(B)** *Claroideoglomus etunicatum*; **(C)** *Acaulospora* sp.

PCA of No AMF treatments allowed us to determine the first principal component (PC1) as accounting for 65.8% of data variation and the second principal component (PC2) for 17.2% of data variation. With PC1 comprising BIO, the treatments did not show significant influence on other variables. On the other hand, in the PC2, comprising No PGPR and Fertilizer D2 (T2), showed more relation with plant height. Inoculation with *Rhizobium* Rh1 without fertilizer (T7) also showed relation with TH (Figure 5A). For inoculation with *C. etunicatum*, PC1 explained 66.2 % of data variation and PC2 19.3 %. PC1 did not show significant relation with other variables. For PC2, the combination of *Rhizobium* Rh1 and No fertilizer (T7) showed more influence on D, TH, and BIO than did other treatments (Figure 5B). When *Acaulospora* sp. was used as inoculum, PC1 grouped 58.9% of data variation and PC2 19.2 %. The treatment with highest impact on plant growth was the combination between *Rhizobium* Rh1 and fertilizer D1 (T9). As for PC2, *Rhizobium* Rh2 plus fertilizer D1 (T12) showed a significant influence on BIO production (Figure 5C).

**Figure 6:**
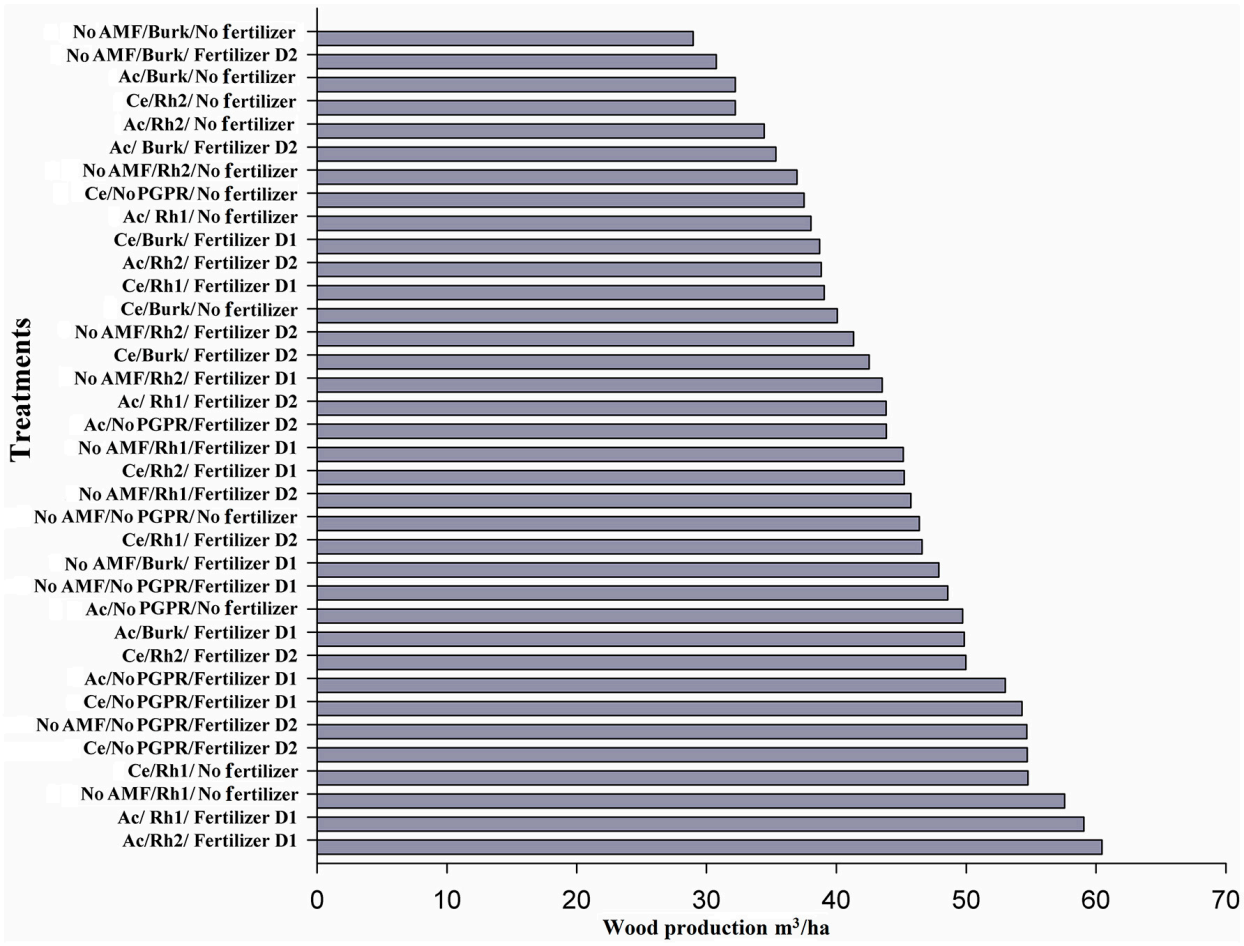
**Wood production by different combinations of AMF [***Acaulospora*** sp. (Ac) and ***Claroideoglomus etunicatum*** (Ce)], PGPR [***Burkholderia*** sp. (Burk), ***Rhizobium*** Rh1, and Rh2], and chemical fertilizer [D1: 75 g plant^**−1**^, D2: 150 g plant^**−1**^] 2 years after seedling planting**.

Wood yield (720 days after seedling planting) increased with *Acaulospora* sp. in combination with *Rhizobium* Rh1 or *Rhizobium* Rh2 strains plus D1. Wood production was around 60 m^3^ ha^−1^ (Figure 6). Single inoculation with *Rhizobium* Rh1 and No AMF/No Fertilizer produced 57 m^3^ ha^−1^ (Figure 6). In this experiment, only two treatments (No AMF/Rh1/No Fertilizer and Ac/Rh1/Fertilizer D1) increased wood production by more than 20% (Figure 6). The Ac/Rh2/Fertilizer D1 treatment resulted in a 30% increase in wood production (Figure 6) when compared to controls. The results showed that wood production varied with inoculation with growth-promoting microorganisms. No PGPR/No Fertilizer produced 46.3 m^3^ ha^−1^, and values for only fertilization with D1 or D2 were 48.5 and 54 m^3^ ha^−1^, respectively (Figure 6).

## Discussion

Several biotic and abiotic factors influence the structural and functional diversity of bacterial communities (Berg and Smalla, 2009). In the relationship between plant and microbial rhizosphere communities, root exudates play an important role in selecting specific microbial populations (Bais et al., 2006). Hence, different plant species are associated with microorganisms that exhibit different responses in terms of survival and activity. In this way, it is necessary to evaluate and select microorganisms from site-specific plant associations, to optimize the inoculant for applications in plant production. The physiological characteristics of the inoculant organism determine to a great extent its fate and activity in soil (Van Veen et al., 1997). In the present study, *Rhizobium* strains promoted the growth of *S. parahyba* var. *amazonicum* when used alone or in combination with *C. etunicatum* or *Acaulospora* sp. in two planting methods. The indigenous isolates of *Rhizobium* were more effective than the exogenous strain of *Burkholderia* sp.

The interaction between AMF and *Rhizobium* improved the development of *S. parahyba* var. *amazonicum* trees from seeds and seedlings. It is believed that the mycorrhiza increase the effectiveness of *Rhizobium* as a result of the general increase in nutritional supply of the host plant (Barea et al., 2002; Bhowmik and Singh, 2004). The ability of *Rhizobium* bacteria to act as endophytes (Spencer et al., 1994; Lupwayi et al., 2004) and PGPR in non-nodulated plants has been confirmed by several studies in other plant species (Yanni et al., 2001; Hossain and Martensson, 2008). Acting as PGPR, rhizobia can support plant growth by solubilizing organic and inorganic phosphates and releasing phytohormones, enzymes, siderophores, exopolysaccharides, and riboflavin (Deshwal, 2013). They can also promote growth by inhibiting the growth of pathogens by the release of antibiotic compounds and/or iron immobilization by siderophore production (Mehboob et al., 2012). The double inoculation of *Rhizobium* and AM fungi has been shown to improve plant growth by increasing the nitrogen and phosphorus contents in plant biomass, resulting in improved soil nutrient availability (Matias et al., 2009).

Mycorrhizal inoculation can be integrated into nursery propagation of forestry species, thereby improving planting performance (Herrera et al., 1993). A more appropriate management of mycorrhizal symbiosis in poor soils would allow substantial reduction in the amount of minerals resulting in minimizing losses in productivity, while at the same time permitting a more sustainable production management (Soka and Ritchie, 2014). Due to the low fertility of the soil in the experimental area (Table 2), application of chemical fertilizers significantly promotes tree growth. This is a common practice in forestry systems in the area, even though this increases the cost of wood production. The main objective of this work was to reduce or improve efficiency of chemical fertilizer application by *in situ* microorganism inoculation of tropical legume trees.

This was demonstrated by the positive effect on plant growth and wood production with the application of combinations of AMF and *Rhizobium*, which were complemented with the addition of low doses of chemical fertilizer, especially in the seed system. The doses applied in the experiment were lower than those reported by Viégas et al. (2007) who used 255–272 g/plant for *S. parahyba* var. *amazonicum* cultivation in the Amazon area. Diameter, height and biomass of *S. parahyba* var. *amazonicum* after 480 days were equal to or greater than values obtained in plants fertilized with the recommended amount of fertilizer, suggesting a favorable and synergistic action between low fertilization and inoculation with *Rhizobium* and/or AMF.

*S. parahyba* var. *amazonicum* shows fast growth, reaching a volumetric production of up to 30 m^3^ ha^−1^ year^−1^ after 6 years of growth (Carvalho, 2007). In this study, the estimation of wood yield with inoculation of microorganisms reached more than 60 m^3^ ha^−1^ in 2 years with the best treatments (Ce/Rh1/Fertilizer D2 in seed sowing and Ac/Rh2/Fertilizer D1 in seedling planting) reaching the maximum yield in 2 years. The promotion of growth of *S. parahyba* var. *amazonicum* by microorganism inoculation has a secondary benefit: carbon sequestration. *S. parahyba* var. *amazonicum* has a low to moderate wood specific density (0.40 g cm^−3^), with carbon representing around 50% of dry matter. The amount of fixed carbon increases when wood production increases.

## Conclusion

The use of microorganisms combined or not with fertilizer was more effective in plant growth and wood production in the seeds experiment as compared to the seedling experiment. Wood yield was almost the same in the two systems. However, when using seeds, many treatments increased plant growth and wood yield, and in the seedling system, only three treatments were more effective compared to control plants.

The use of native microorganisms as an inoculant for S. *parahyba* var. *amazonicum* was very effective, especially when combined with low doses of fertilizer, resulting in increased plant growth and wood yield under field conditions. In addition, the inoculation of *Acaulospora* sp. and bacteria improved the absorption of chemical fertilizer, enhancing wood yield. When compared with non-fertilized trees, the best treatments increased wood production by more than 50%. The inoculation of *Acaulospora* sp. and *Rhizobium* Rh1 with D1 fertilizer was the most effective treatment in both systems.

## Author contributions

MC: Experimental design and collect of data in the field. MS: Plant culture in the field. JE, FS, VF, AB, EG, GL: Field team control pest, average of plants in the field. IS, AO: Statistical analysis. GA: General coordinator, Head leader team.

### Conflict of interest statement

The authors declare that the research was conducted in the absence of any commercial or financial relationships that could be construed as a potential conflict of interest.
